# Claudin-1 Is a p63 Target Gene with a Crucial Role in Epithelial Development

**DOI:** 10.1371/journal.pone.0002715

**Published:** 2008-07-23

**Authors:** Teresa Lopardo, Nadia Lo Iacono, Barbara Marinari, Maria L. Giustizieri, Daniel G. Cyr, Giorgio Merlo, Francesca Crosti, Antonio Costanzo, Luisa Guerrini

**Affiliations:** 1 Department of Biomolecular Sciences and Biotechnology, University of Milan, Milan, Italy; 2 Department of Dermatology, University of Rome “Tor Vergata”, Rome, Italy; 3 INRS-Institut Armand-Frappier, Laval, Quebec, Canada; 4 Dulbecco Telethon Laboratory, Molecular Biotech Center, University of Torino, Torino, Italy; 5 Medical Genetics Laboratory A.O. San Gerardo, Monza, Italy; University of Hong Kong, China

## Abstract

The epidermis of the skin is a self-renewing, stratified epithelium that functions as the interface between the human body and the outer environment, and acts as a barrier to water loss. Components of intercellular junctions, such as *Claudins*, are critical to maintain tissue integrity and water retention. p63 is a transcription factor essential for proliferation of stem cells and for stratification in epithelia, mutated in human hereditary syndromes characterized by ectodermal dysplasia. Both *p63* and *Claudin-1* null mice die within few hours from birth due to dehydration from severe skin abnormalities. These observations suggested the possibility that these two genes might be linked in one regulatory pathway with p63 possibly regulating *Claudin-1* expression. Here we show that silencing of ΔNp63 in primary mouse keratinocytes results in a marked down-regulation of *Claudin-1* expression (−80%). ΔNp63α binds *in vivo* to the *Claudin-1* promoter and activates both the endogenous *Claudin-1* gene and a reporter vector containing a –1.4 Kb promoter fragment of the *Claudin-1* gene. Accordingly, *Claudin-1* expression was absent in the skin of E15.5 *p63* null mice and natural p63 mutant proteins, specifically those found in Ankyloblepharon–Ectodermal dysplasia–Clefting (AEC) patients, were indeed altered in their capacity to regulate *Claudin-1* transcription. This correlates with deficient *Claudin-1* expression in the epidermis of an AEC patient carrying the I537T *p63* mutation. Notably, AEC patients display skin fragility similar to what observed in the epidermis of *Claudin*-1 and *p63* null mice. These findings reinforce the hypothesis that these two genes might be linked in a common regulatory pathway and that *Claudin-1* may is an important *p63* target gene involved in the pathogenesis of ectodermal dysplasias.

## Introduction

To maintain homeostasis in multicellular organisms the isolation and compartmentalization of the internal environment is essential and is achieved by organization of ectodermal tissue in multiple cellular sheets. Epithelial sheet architecture is dynamically maintained through the combined action of tight junctions, adherens junction and desmosomes. Of these, adherens junction proteins, such as E-cadherin, and desmosomes are primarily responsible for the adhesion between adjacent cells, whereas tight junctions regulate permeability and the paracellular exchange of water, ions, and macromolecules across epithelial sheets [1], [2]. The Tight Junction (TJ) family comprises three main classes of proteins: claudins, occludin and junctional adhesion molecules. Claudins and occludins build up the functional units responsible for the tight sealing of the cells in epithelial sheets, whereas TJ proteins, such as Tight Junction Protein 1 (TJP1), are responsible for linking claudins and occludin to the actin cytoskeleton [3]–[5]. Claudins are directly involved not only in the formation of TJ strands but also in their barrier function in simple epithelia.

Recent advances revealed that claudins are directly involved in intercellular sealing of simple as well as stratified epithelia in vertebrates.

Claudins expressing genes comprise a large family consisting of at least 24 members in mice/humans [6]–[9]. When claudins are expressed singly in fibroblasts lacking TJs, well-developed networks of TJ strands are organized between adjacent transfectants [10]. More than two claudin species are often co-expressed in single cells of various tissues.

Recently, *Claudin-1* (*Cldn-1*) null mice have been generated; these mice show severe skin abnormalities and die of dehydration within one day of birth. Although the layered organization of keratinocytes appeared to be normal, the epidermal barrier was severely affected in these mice [11].

The epithelium organization and the dehydration defects observed in *Cldn-1* null mice are similar to those observed in *p63* null mice. Indeed, mice in which *p63* was inactivated failed to develop stratified epithelia and epithelial appendages, such as teeth, hair follicles and mammary glands and died within one day of birth from dehydration, as did the *Cldn-1* null mice [12], [13]. The *Tp63* gene encodes a transcription factor homologous to the p53 tumor suppressor, which is consistently expressed in basal cells of stratified epithelia [14]. Unlike p53, p63 does not function as a classical tumor-suppressor; instead, it functions primarily in epithelial-mesenchymal development during embryogenesis.

Multiple products are produced from the *p63* gene. The use of alternative promoters drives transcription of TAp63 proteins, having an aminoterminal Trans Activation (TA) domain, a DNA Binding (DB) domain and an Oligomerization Domain (OD), or ΔNp63 proteins lacking the transactivation domain homologous to that of p53 [15]. However, additional TA domains have been identified that account for the transcriptional activities of the ΔN isoforms [16]–[18]. In addition three alternative splicing routes at the 3′ end generate proteins with different C-termini, denoted α, β and γ. A Sterile Alpha Motif (SAM) is contained only in the α-isoforms (TA and ΔN). The presence of a SAM, which is absent in p53, is the most significant structural difference between p63 and p53 [19]. SAM domains are protein-protein interaction domains also found in other developmentally important proteins, such as p73 and several Eph receptor tyrosine kinases [20].

Similarities between the *Cldn-1* and *p63* null mice suggested the possibility that these two genes might be linked to the same regulatory pathway or belong to a common signaling cascade with p63 possibly regulating *Cldn-1* expression.

Here we report that: 1) *Cldn-1* expression is severely reduced upon siRNA mediated downregulation of *ΔNp63* isoforms, 2) *Cldn-1* expression is absent in the skin of E15.5 *p63* null mice, 3) the ΔNp63α isoform binds to the *Cldn-1* promoter in keratinocytes *in vivo*, 4) a 1.4 Kb *Cldn-1* promoter fragment is activated by ΔNp63α, 5) natural ΔNp63 mutations, in particular associated with the AEC syndrome which displays the most severe skin phenotype among p63 associated syndromes, were unable to activate *Cldn-1* transcription, and 6) a skin biopsy derived from an AEC patient confirmed the *in vitro* evidences, with a clear and sharp reduction of Cldn-1 expression in the basal layer of the epidermis.

These results indicate that *Cldn-1* is an important p63 target gene needed for normal skin development and for the maintenance of barrier function of the skin.

## Materials and Methods

### Plasmids

Expression vectors encoding all mouse *p63* cDNAs in the pcDNA3 expression vectors have been described [16]. The *Cldn-1* promoters have also been previously described [21].

### Cell cultures and transfection

Primary mouse keratinocytes were isolated from newborn mice and cultured at 37°C in low calcium (0.05 mM CaCl_2_) keratinocyte basal medium (KBM, Clonetics, San Diego, CA) and EGF (10 ng/ml). Mouse primary keratinocytes were induced to differentiate by CaCl_2_ (2 mM) treatment for 24 hours. Human lung carcinoma H1299 cells stably transfected with a tet-on ΔNp63α expression plasmid, were grown and induced with Doxycyclin, as previously described [22].

The human osteosarcoma U2OS cell line was maintained in Dulbecco's modified Eagle's medium (D-MEM) and 10% fetal calf serum. For transfection, 50,000 cells were seeded into 24-well multi-plates and on the next day transfected with Lipofectamine 2000 (Invitrogen) according to the manufacturer's instructions. The total amount of transfected DNA (1 µg) was kept constant using empty vector when necessary. Twenty-four hours later, cells were lysed and assayed for luciferase activity.

### RNA interference and analysis

The scrambled- siRNA (Dharmacon Research) was used as negative control. siRNA duplexes and targeting ΔNp63 and GFP were obtained from MWG-Biotech (Ebersberg, Germany).

The sense strands of the siRNAs used in this study were as follows:

-GFP (ctr) siRNA 5′-GTTCAGCGTGTCCGGCGAG-3′

-ΔNp63 siRNA 5′-TGCCCAGACTCAATTTAGT-3′

Primary mouse keratinocytes, plated on collagen-coated 35 mm dishes were transfected with 0.5 µg siRNA/dish using Lipofectamine 2000 (Invitrogen). 24 hours after transfection total mRNA was extracted, subjected to retrotranscription and to RealTime PCR using specific primers.

### Semiquantitative RT-PCR and RealTime PCR

Total RNA was prepared from H1299 cells and from primary mouse keratinocytes with TRIzol reagent (Sigma). 1 µg of total RNA was retrotranscribed using Super Script II MMLV enzyme (Invitrogen). RealTime PCR was performed on mouse primary keratinocytes and on mouse ectoderm from Fore Limbs (FLs) and Hind Limbs (HLs) as previously described [23].

The following oligonucleotides were used for gene amplification:[Table pone-0002715-t001]

**Table pone-0002715-t001:** 

***h-CLDN-1***			
Forward	5′	ACTCCTTGCTGAATCTGAACAGT	3′
Reverse	5′	GGACACAAAGATTGCGATCAG	3′
***h-GAPDH***			
Forward	5′	TCACCAGGGCTGCTTTTAAC	3′
Reverse	5′	TGGAAGATGGTGATGGGATT	3′
***m-CLDN-1***			
Forward	5′	ACTCCTTGCTGAATCTGAACAGT	3′
Reverse	5′	GGACACAAAGATTGCGATCAG	3′
***m-CLDN-3***			
Forward	5′	CTCATCGTGGTGTCCATCC	3′
Reverse	5′	ATGGTGATCTTGGCCTTGG	3′
***m-CLDN-10***			
Forward	5′	AGCTTCTCTGCATCATTGG	3′
Reverse	5′	TTCTCCGCCTTGATACTTGG	3′
***m-*** **GAPDH**			
Forward	5′	TGTCAGCAATGCATCCTGCA	3′
Reverse	5′	TGTATGCAGGGATGATGTTC	3′
***m-CyclophilinA***			
Forward	5′	ATGGTCAACCCCACCGTGTT	3′
Reverse	5′	CGTGTGAAGTCACCACCCT	3′
***m-Tp53***			
Forward	5′	TCTACAAGAAGTCACAGCACATGAC	3′
Reverse	5′	CCTTCCACCCGGATAAGATGC	3′
***m-ΔNp63***			
Forward	5′	ATGTTGTACCTGGAAAACAATG	3′
Reverse	5′	GATGGAGAGAGGGCATCAAA	3′

### Immunoblot analysis and antibodies

Twenty-four hours after transfection, cells were lysed in 100 µl of loading buffer (2% sodium dodecyl sulfate, 30% glycerol, 300 µM β-mercaptoethanol, 100 mM Tris-HCl pH 6.8): extracts were separated on SDS-10% polyacrylamide gels and transferred to a nitrocellulose membrane (Scleicher & Schuell). The blots were incubated with the Cldn-1 antibody (Zymed) or the 4A4 p63 antibody (Santa Cruz Biotech) and developed according to the manufacturer's instructions (Super Signal, Pierce).

### Chromatin ImmunoPrecipitation (ChIP)

Mouse primary keratinocytes uninduced or induced to differentiate for 24 hours were washed in PBS, incubated for 10 minutes with 1% formaldehyde and quenched with Glycine 0.1 M. Cells were sonicated and chromatin fragments of an average length of 0.5 Kb recovered by centrifugation. Immunoprecipitations were performed with ProtG-Sepharose (KPL, USA), blocked twice at 4°C with 1 µg/ml salmon sperm DNA sheared at 500 bp length and 1 µg/ml BSA, for 2 hours and overnight. Chromatin was precleared by adding ProtG-Sepharose for 2 hours at 4°C, aliquoted and incubated with 3–5 µg of p63 antibodies overnight at 4°C. DNA was released by incubating samples for 5 hours at 65°C, phenol-extracted and ethanol precipitated. Semiquantitative PCR was performed with the following primers annealing to the mouse *Cldn-1* regulatory region:[Table pone-0002715-t002]

**Table pone-0002715-t002:** 

R1- Forward	5′	GGGATGCCTGATTCTCTTCA	3′
R1- Reverse	5′	TGGAAGTGAGAAGTGGCAGA	3′
R2- Forward	5′	TGGAAGCATCCCTTGTTTTC	3′
R2- Reverse	5′	TTGCTGTCCTCTCTGGGTCT	3′
R3- Forward	5′	CTGAACAGAGGCCATCCCTA	3′
R3- Reverse	5′	CACGTAGTCTGGCACACACA	3′

As control, oligonucleotides annealing to the *Ikkα* mouse promoter were used:[Table pone-0002715-t003]

**Table pone-0002715-t003:** 

Forward	*5*′	CTCAAGTCGGAGAACAAGG	3′
Reverse	5′	CTCGAATCCTACCACACTCG	3′

### Preparation of tissue samples and *in situ* hybridization

Experiments involving the use of animals were approved by the Institutional Animal Care Committee and by the Ministry of Health. Pregnant females (*p63^−/+^*×*p63^−/+^*) [13] were sacrificed at Embryonal day 15.5 (E15.5) of gestation, epidermal-dermal tissues were dissected from the embryonic flank, fixed in PFA 4%, cryoprotected in 20% sucrose, embedded in OCT and sectioned at 11 µm. *In situ* hybridization was carried out with DIG-labeled antisense RNA probes, according to published procedures [24]. The *Claudin-1* probe (399 bp in length) corresponds to the coding sequence and part of the 5′ UTR of the murine mRNA. The *p63* probe (497 bp in length) corresponds to exons 4-11 of murine *p63* mRNA, common to both the *TA* and the *ΔN* transcripts.

### Immunohistochemistry

Four-mm punch biopsy specimens were taken from normal skin of healthy volunteers (n = 4). The declaration of Helsinki protocols were followed and the patient parents gave written approved consent before biopsy. A patient affected by AEC (I537T mutation in *p63*) was subjected to skin biopsy on lesions after obtaining informed consent from his parents. Paraffin embedded 5 mm skin sections were kept at 60°C for 1 hour, and then incubated at 80°C overnight in citrate buffer pH6 (DAKO Italia, Milan, Italy). The sections were incubated for 1 hour at room temperature with the appropriate dilution of anti-human Cldn-1 monoclonal antibody (Zymed), anti-p63 (4A4, Santa Cruz Biotec) or control mouse IGgs. Sections were stained with an avidin-biotin-peroxidase technique (DAKO Milan, Italy) by using 3,3′-diaminobenzidine as a substrate (DAKO Milan, Italy).

## Results

### *Cldn-1*, *3* and *10* are downregulated in ΔNp63 deficient keratynocites

**Figure 1:**
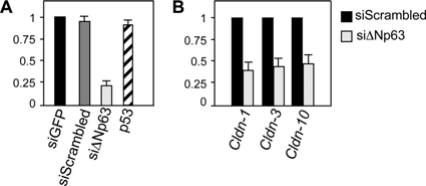
*Cldn-1* expression is down-regulated upon siRNA to *ΔNp63*. A) Primary mouse keratinocytes were transfected by control siRNA (siGFP), scrambled siRNA (Dharmacon) or *ΔNp63* specific siRNA (siΔNp63). 24 hours after transfection total RNA was extracted and subjected to RealTime PCR to verify *ΔNp63* and *p53* downregulation. Results are expressed as mean expression of three different samples. Standard Deviations (SD) are indicated. B) *Cldn-1*, *3* and *-10* expressions in the same samples as in Figure 1A. Expression of the three genes was normalized to *CyclophilinA* expression. SD from three different samples is indicated.

Defects in the barrier function of the skin are evident in both *p63* null mice and in *p63*-dependent ectodermal dysplasias, such as in the AEC syndrome. In order to determine the pattern of genes controlled by p63 and involved in the control of skin development and of barrier function, we have selectively downregulated *ΔNp63* isoforms in primary mouse keratinocytes by means of specific siRNAs (siΔNp63) [25]. The expression of *ΔNp63* upon siΔNp63 was reduced by 80% while the expression of p53 did not change (Figure 1A). As shown in Figure 1B, *ΔNp63* downregulation caused a strong decrease in the expression of *Cldn-1 -3* and *10*, identifyng these genes as potential mediators of ΔNp63 control over barrier function in adult skin.

Given the well known role played by Cldn-1 in the formation of TJs, we decided to focus on its regulation by p63.

### ΔNp63_α functionally interacts *in vivo* with two different regions of the *Cldn-1* gene

**Figure 2:**
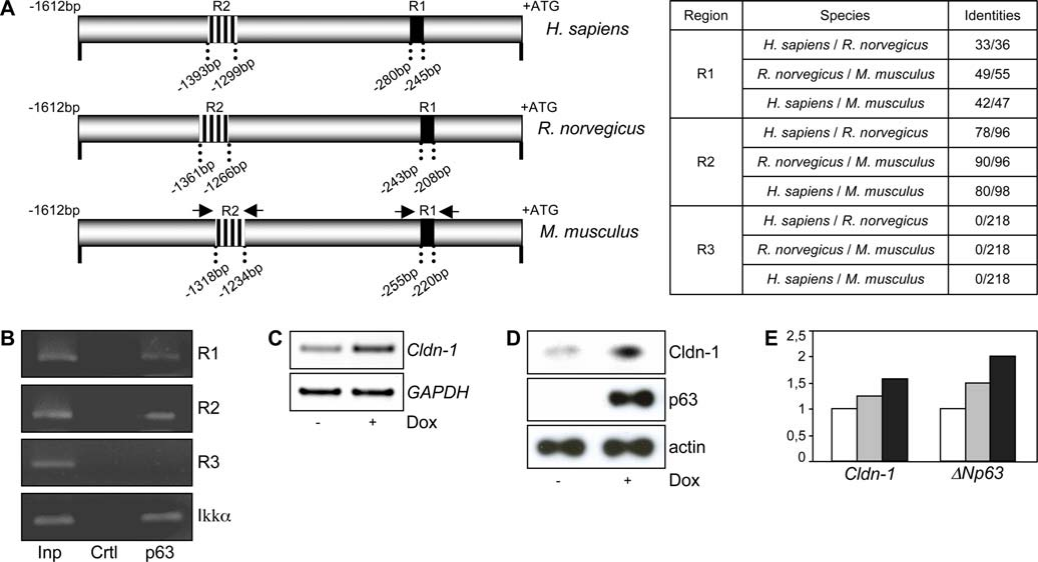
ΔΝp63_α functionally interacts *in vivo* with the *Cldn-1* gene. A) Schematic representation of the human, rat and mouse *Cldn-1* promoter regions. Two regions of high interspecies homology were identified (R1 and R2, black and striped boxes respectively). The degree of homology within R1 and R2 is shown on the right. R3, mapping at −3.5 Kb from the ATG of the *Cldn-1* gene, did not show any homology and was used as negative control. B) Three amplicons centered on R1, R2 and R3 of the *Cldn-1* gene were used in semiquantitative PCR amplifications with chromatin extracted from mouse primary keratinocytes, induced with Ca^2+^ for 24 hours, and immunoprecipitated with anti-p63 antibodies (4A4, Santa Cruz Biotech). R1 and R2 were positives with the anti-p63 antibodies while R3 was negative. As positive control, oligonucleotides annealing to the *IKKα* mouse promoter [25] were used. C) H1299 cells were treated with 20 µM Doxicycline in order to induce ΔNp63α expression. Thirty hours after induction mRNA was extracted from uninduced (-Dox) and induced (+Dox) cells and levels of endogenous *Cldn-1* and *GAPDH* were assessed by semiquantitative RT-PCR. D) Expression of the Cldn-1 protein is clearly detected upon ΔNp63α expression in H1299 cells. E) RNA extracted from mouse HL at E10.5, E11.5 and E12.5 (white, grey and black bars respectively) were used to verify *ΔNp63* and *Cldn-1* levels of expression.

To study the potential role of p63 as a direct transcriptional regulator of *Cldn-1*, we performed Chromatin Immuno Precipitation (ChIP) experiments to verify its direct binding to the *Cldn-1* promoter. To this aim, we used chromatin extracted from primary mouse keratinocytes, endogenously expressing ΔNp63α, placed under differentiating conditions by Ca^2+^ addition; chromatin was immunoprecipitated with anti-p63 antibodies. We scanned the *Cldn-1* promoter with three pairs of primers; two of them were designed on two regions of high homology between rat, mouse and human promoters (Region 1 and Region 2, R1 and R2) while the third one was designed on a region that did not show any homology (Region 3, R3), as shown in Figure 2A. In Figure 2B, the results showed that of the three amplicons used, the ones corresponding to R1 and R2 of high homology between human, mouse and rat, were positive for p63 binding.The R3 was negative for p63 binding.

In order to verify whether or not the binding of ΔNp63α to the endogenous *Cldn-1* gene was associated to changes in *Cldn-1* gene expression, we used a cell line derived from the human H1299 cell line, stably transfected with a Doxicycline inducible ΔNp63α [22]; this cell line was used to quantify the levels of *Cldn-1* specific transcript as well as Cldn-1 protein expression upon ΔNp63α induction. To this aim total RNA and proteins were extracted and used in semiquantitative RT-PCR reactions with oligonucleotides specific for the *Cldn-1* transcript and in western blot analysis with anti Cldn-1 antibodies. Expression of endogenous *Cldn-1* was activated in response to ΔNp63α over-expression both at the mRNA and protein levels (Figure 2C and 2D).

We have recently shown that the expression of the ΔNp63 isoforms increases during mouse limb development [23]: we determined *Cldn-1* expression in RNA samples extracted from E10.5, E11.5 and E12.5 Hind Limb (HL) and Fore Limb (FL). As shown in Figure 2E, there is a concomitant increase of *ΔNp63* and *Cldn-1* expression at E12.5 in the HL (FL, data not shown).

**Figure 3:**
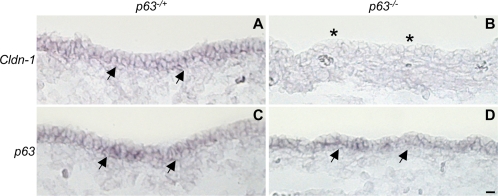
Reduced *Cldn-1* expression in the skin epithelium of *p63* null embryos. Sections through the skin epithelium and dermis of *p63^−/+^*(A and C) and *p63^−/−^* (B and D) embryos, hybridized with *Cldn-1* (A and B) and with *p63* (A and B) probes. Black arrows, hybridization signal on epithelial cells. Black asterisks in B, absence of signal. Scale bar (D, bottom right), 10 µm.

Finally, we examined the expression of *Cldn-1* in the skin of E15.5 *p63* null embryos [13], by *in situ* hybridization. *Cldn-1* expression was detectable in the skin epithelium of *p63^−/+^* embryos, while absent in *p63^−/−^* ones (Figure 3A and B). As a control for RNA preservation, consecutive sections were hybridized with a probe that detects both *TA* and *ΔNp63* transcripts. In fact, in the *p63^−/−^* mice, the *p63* mRNA is still expressed from the targeted allele, while no protein is being made [13]. *p63* hybridization signal was detected in both normal and *p63* null skin, as expected (Figure 3C and D).

All together, these data indicate that p63 expression is necessary for Cldn-1 expression in the skin epithelium and that upon ΔNp63α expression, p63 is functionally associated *in vivo* to the *Cldn-1* gene and that this association enhances the transcription from the *Cldn-1* gene.

### A −1.4 Kb fragment of the *Cldn-1* promoter is regulated by ΔNp63 isoforms

**Figure 4:**
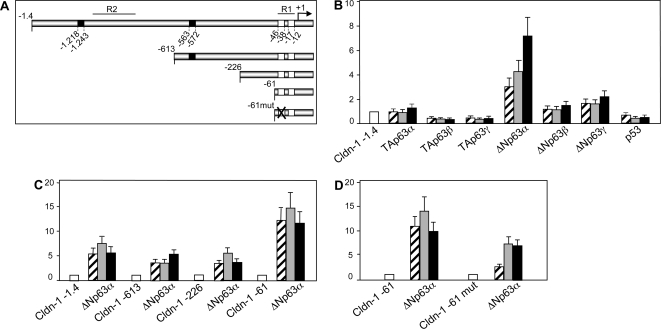
The *Cldn-1* promoter is regulated by the ΔNp63_ isoforms. A) Schematic representation of the *Cldn-1* promoter constructs used. White boxes: *Sp1* binding sites. Black boxes: *p53* binding sites. B) U2OS cells were transfected with the −1.4 Kb *Cldn-1* reporter plasmid (0.3 µg) (white bar). Different quantities of expression plasmids for the p63 isoforms and p53 were cotransfected (0.025, 0.05 and 0.1 µg) (striped, light grey and black bars respectively). C) U2OS cells were transfected with the deletion constructs of the *Cldn-1* reporter plasmid (0.3 µg). Different quantities of expression plasmids for the ΔNp63α were cotransfected (0.025, 0.05 and 0.1 µg) (striped, light grey and black bars respectively). D) U2OS cells were transfected with the wild- type or the mutated −61 bp promoter constructs (0.3 µg). Different quantities of expression plasmids for the ΔNp63α were cotransfected (0.025, 0.05 and 0.1 µg) (striped, light grey and black bars respectively). Cells were lysed after 24 hours and luciferase activity was determined. The basal activity of the reporter plasmids was set to 1. Data are presented as fold activation/repression relative to the sample without effectors. Each bar of the histogram represents the mean of three independent transfection duplicates. Standard deviations are indicated.

The results of the ChIP analysis prompted us to tackle a functional analysis of the *Cldn-1* promoter. To further study the regulation of *Cldn-1* expression by p63 we employed a luciferase reporter plasmid containing a -1.4 Kb fragment of the rat *Cldn-1* regulatory region [21], that contains both conserved R1 and R2 (Figure 2A). This fragment contains also two putative *p53* binding sites which location however did not overlap with R1 and R2 (Figure 4A). We performed transient transfections in the U2OS cell line that do not express p63 and express p53; we systematically co-transfected different doses of plasmids encoding all p63 isoforms with the −1.4 Kb *Cldn-1* promoter in subsequent experiments. As shown in Figure 4B, in the U2OS cells the *Cldn-1* promoter activity was repressed by the co-transfection of serial doses of TAp63β and TAp63γ expression plasmids while TAp63α was inactive. On the other hand, co-transfection of the ΔNp63α isoform transactivated this promoter while ΔNp63β and ΔΝp63γ isoforms had little effect on this promoter. p53 transfection repressed the activity on the *Cldn-1* promoter. We repeated the same experiments in the Saos-2 cell line, expressing no p53 and no p63 endogenously, and obtained similar results (data not shown).

In order to identify the region of the *Cldn-1* promoter responsible for the observed regulation by ΔNp63α, we employed serial deletion constructs of the *Cldn-1* promoter [21] in co-transfection experiments with the ΔNp63α isoform in the U2OS cells. The results (Figure 4C) clearly showed that deletion of a region encompassing −1.4 kb and −613 bp of the *Cldn-1* promoter, deleting R2 and one of the two putative *p53* binding sites, resulted in a reduction of transactivation by ΔNp63α and a further deletion up to −226 bp retained a similar activity to the −613 construct. Strinkingly, a deletion up to −61 bp, shown to be necessary and sufficient for Cldn-1 expression in rat epididymal cells [21], showed high transactivation by ΔNp63α. Within these 61 bp, the R1 is contained; two *Sp1* binding sites have been identified, exactly within R1, and mutation of the distal *Sp1* site resulted in a 4 fold reduction of Cldn-1 basal activity [21]. Since p63 has been shown to physically and functionally interact with Sp1 [26], [27], we employed a version of the *Cldn-1* promoter mutated in the *Sp1* distal site in co-transfection with ΔNp63α: mutation of the distal *Sp1* binding site reduced ΔNp63α dependent transactivation of the *Cldn-1* promoter, suggesting that indeed the activation observed in the −61 bp fragment could be the result of p63 interacting with Sp1 (Figure 4D).

These data indicate that the ΔNp63α isoform is the main inducer of the *Cldn-1* promoter and that two regions in the *Cldn-1* promoter are mediating this activity: the first one contained within −1.4 kb and −613 bp where the R2 of high interspecies homology is contained and where p63 binds in ChIP experiments; the second one, where p63 also binds, containing the R1 and two *Sp1* binding sites; the distal *Sp1* binding sites seems to mediate part of p63 action.

### The AEC natural p63 are loss of function mutation for *Cldn-1* promoter activity

**Figure 5:**
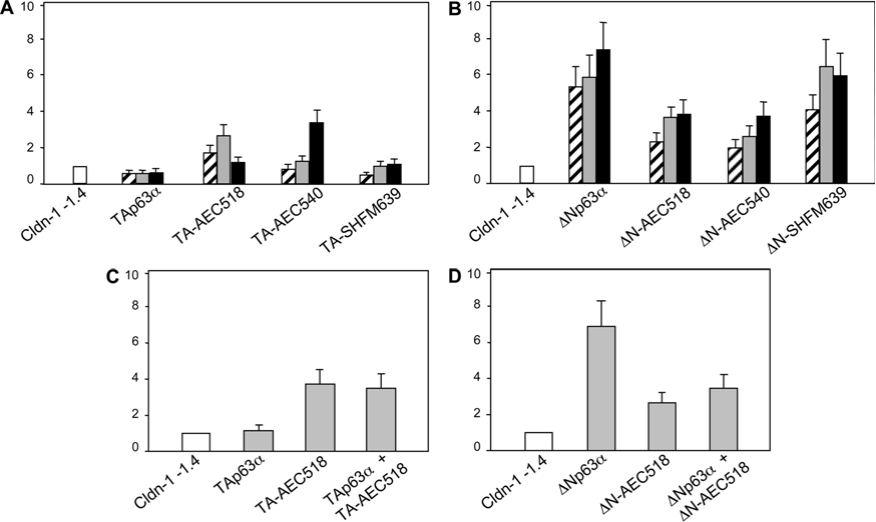
The AEC mutations subvert p63 transcriptional potential. A) U2OS cells were transfected with the −1.4 Kb *Cldn-1* reporter plasmid (0.3 µg) (white bar). Different quantities of expression plasmids for TAp63 mutants were cotransfected (0.025, 0.05, 0.1 µg) (striped, light grey and dark grey bars respectively). B) U2OS cells were transfected with the −1.4 Kb *Cldn-1* reporter plasmid (0.3 µg) (white bar). Different quantities of expression plasmids for ΔNp63 mutants were cotransfected (0.025, 0.05, 0.1 µg) (striped, light grey and dark grey bars respectively). C) 0.05 µg of TAp63α or TA-AEC518 were transfected either alone or together in order to reproduce the heterozygous state found in AEC patients. D) 0.05 µg of ΔNp63α or ΔN-AEC518 were transfected either alone or together in order to reproduce the heterozygous state found in AEC patients. Cells were lysed after 24 hours and luciferase activity was determined. The basal activity of the reporter plasmids was set to 1. Data are presented as fold activation/repression relative to the sample without effectors. Each bar of the histogram represents the mean of three independent transfection duplicates. Standard deviations are indicated.

The phenotypic similarity between the *Cldn-1* and *p63* null mice, and the phenotype of patients affected by syndromes associated with *p63* mutations, prompted us to analyze the possibility that natural *p63* mutations (specifically those associated with ectodermal defects in human) affect the transcriptional function of p63 on the *Cldn-1* promoter. We used *p63* mutations associated to two human syndromes, AEC and Split Hand Foot Malformation type IV (SHFM-IV): in AEC patients severe skin abnormalities are observed that are absent in SHFM-IV patients. Co-transfection experiments in the U2OS cell line, clearly showed that the TA-AEC518 and TA-AEC540 mutants had gained activation potential compared to the TA-SHFM639 mutant and wild type TAp63α that did not possess this activity (Figure 5A). On the other hand, ΔN-AEC518 and ΔN-AEC540 mutants had a reduced transactivation potential while the activation potential of the ΔN-SHFM639 mutant was very similar to that of wild type ΔNp63α (Figure 5B).

In patients, *p63* mutations are always occurring as dominant heterozygous mutations. In order to mimic the heterozygous state found in patients *in vivo*, we performed co-transfection experiments of ΤΑp63α with TA-AEC518 and ΔNp63α with ΔN-AEC518 (1∶1 ratio). As shown in Figure 5C, co-transfection of ΤΑp63α with TA-AEC518 resulted in incresead levels of transactivation compared to that obtained TAp63α alone. On the other hand, co-transfection of ΔN-AEC518 with ΔNp63α reduced the transactivation obtained with ΔNp63α alone (Figure 5D).

These data show that *Cldn-1* expression is profoundly affected by *p63* mutations associated to the AEC syndrome and raises the possibility that *Cldn-1* expression may also be altered in the skin epithelium of AEC patients.

### Reduced expression of Cldn-1 in AEC skin

Based on the data presented thus far, we hypothesize that Cldn-1 may be involved in the pathogenesis of skin defects observed in AEC syndrome. To verify this hypothesis *in vivo* we examined Cldn-1 expression in skin lesions of a patient with a severe form of AEC syndrome associated to the presence of the I537T mutation in one of the *p63* allele (A. Costanzo, unpublished data), a mutation already described in other cases of AEC cases [19].

**Figure 6:**
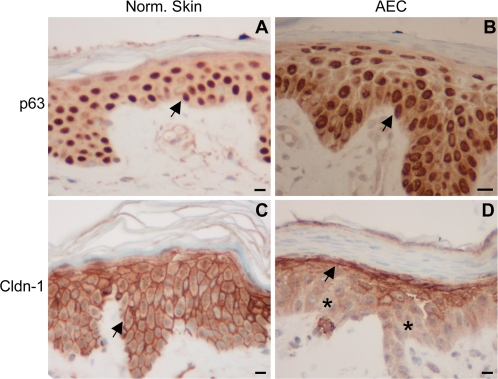
The I537T AEC mutation alter Cldn-1 expression *in vivo*. Skin sections from healthy donor (A and C) and from lesional skin of a patient affected by AEC syndrome (I537T mutation in the SAM domain of p63) (B and D) were subjected to immunohistochemistry with specific antibodies to reveal the pattern of expression of p63 (A and B) and Cldn-1 (C and D). Black arrows, immunohistochemistry signal on epithelial cells. Black asterisks in D, absence of signal. Black bars indicate 10 µm.

We carried out show immunohistochemical staining to detect p63 and Cldn-1 expression in normal skin compared to AEC lesional skin. While p63 expression was equally distributed in both the normal and the AEC skin (Figure 6, panels A–B), we observed a profound change in Cldn-1 expression pattern in the AEC skin. In normal skin Cldn-1 was strongly expressed on the membrane of keratinocytes throughout the epidermis layers, while in the AEC epidermis we were unable to detect Cldn-1 expression in the basal and suprabasal layers and observed only a weak positivity on the membrane of few cells of the granular layer (Figure 6, compare panels C–D). Thus, Cldn-1 is strongly down-regulated in the AEC skin; this observation suggests that *Cldn-1* may belong to the set of p63 target genes whose modification causes skin fragility in AEC patients.

## Discussion

The skin is a self-renewing, stratified epithelium that functions as the interface between the human body and the outer environment. The epidermal layer not only protects us from environmental pathogens but also acts as a ‘barrier’ to water loss. The molecules regulating this important function are components of intercellular junctions, termed TJ, which play an essential role in development of barrier function in the skin.

Data derived from the analysis of TJ structure and null mice phenotype indicate that Cldn-1 is required for TJ barrier function to prevent water loss [11]. Defects in skin barrier function are present in several skin diseases including inflammatory and genetic diseases.

We have found that Cldn-1, the principal component of TJs, is a downstream target of the p63 transcription factor, essential for proper epidermal development.

p63 controls several aspects of stratified epithelia development and homeostasis acting through the transcriptional regulation of a definite set of genes which contribute to proliferation, differentiation and cell-cell adhesion in stratified epithelia [28]. Loss of *p63* expression in null mice leads to death within 24 hours after birth because of massive water loss, suggesting that the TJs function is lost in the skin of these mice.

Our data indicate that the ΔNp63α isoform, which is expressed in ectodermal cells concomitantly with the activation of the stratification program in mouse embryo and throughout adult stratified epithelia, is a direct transcriptional activator of *Cldn-1*. *Cldn-1* activation by p63 seems to be isoform specific, being ΔNp63α the best activator and TAp63 isoforms mild repressors. ΔNp63α seems to play a major role in regulating expression of the *Cldns* family; infact, we have observed down regulation of *Cldn-1*, *Cldn-3* and *Cldn-10* in cells transfected with siRNA targeting *ΔNp63* and *Cldn-1* was undetectable in the skin of *p63* null mice, further supporting the evidences that *Cldn-1* expression is dependent on p63 expression.

A complex regulation has been shown to occur for the Cldns. Studies on compensatory responses of different Cldns appear to be tissue specific. Studies in MDCK cells using siRNA failed to show any compensatory regulatory mechanisms following the down regulation of Cldns 2-4 and Cldn-7 [29]. Others have reported that overexpression of Cldn-2, -7, -15 did not alter the expression of other Cldns in Caco cells [30]–[32]. In contrast, Cldn-12 overexpression in Caco cells resulted in an increased expression of Cldn-2. Yu et al. have also reported that overexpression of Cldn-8 results in the downregulation of Cldn-2 [33]. Gow et al. reported increased expression of Cldn-3 in basal cells of the cochlea of *Cldn-1* null mice, however, other Cldns were unaffected [34].

Using bioinformatic analysis, we identified two regions (R1 and R2) of high sequence homology between the rat, mouse and human *Cldn-1* promoter regions. Both R1 and R2 are bound by ΔNp63α in keratinocytes induced to differentiate. The observation of ΔNp63 recruitment on *Cldn-1* promoter upon keratinocyte differentiation suggests that *Cldn-1* may be part of an important set of genes, such as *IKKα* [25], which are specifically activated by ΔNp63 and required for induction of correct stratification program. Moreover, both R1 and R2 are necessary to achieve the full activation by ΔNp63, as determined by luciferase transcriptional assays (Fig. 4C).

In the *Cldn-1* promoter the bioinformatics analysis identified two *p53* binding sites; these two sites did not however overlap with R1 and R2 and p53 had a repressive effect on *Cldn-1* promoter activity. The first *p53* binding site, located at −1.2 Kb of the *Cldn-1* promoter, is quite close to the R2 region (Figure 4A) and, due to the average size of the chromatin used in the ChIP analysis, we cannot exclude that p63 is indeed binding to this site. The second *p53* binding site, located at −0.5 Kb, doesn't seems to play a role in ΔNp63α mediated transactivation since its deletion did not have any effect on ΔNp63α mediated transactivation of the *Cldn-1* promoter (compare the −613 bp and −226 bp constructs, Figure 4C).

The −61 bp construct showed very high levels of transactivation by ΔNp63α; this can be explained by the deletion of the −125 to −61 region that has been shown to repress *Cldn-1* promoter activity [21]. The R1 is contained within the −61 bp construct where two *Sp1* binding sites have been identified [21]. Sp1 binding to the distal *Sp1* site has been shown to be important for basal activity of the *Cldn-1* promoter in epididymal cells [21]. p63, as p53, can bind DNA directly or can interact with other transcription factors already bound to the DNA. p63 has been shown to physically and functionally interact with components of the basal transcriptional apparatus such as NF-Y [35] and Sp1 [26], [27]. Mutation of the *Sp1* distal site in the *Cldn-1* promoter reduced ΔNp63α dependent transactivation, suggesting that the observed activation by ΔNp63α on the -61 bp *Cldn-1* promoter construct could be the result of p63 interacting with Sp1. A finer analysis of the *Cldn-1* promoter will help to clarify these points.

Dominant mutations in the *p63* gene cause a specific subset of syndromes characterized by various degrees of skin/hair defects and by other phenotypes including craniofacial and limb malformations, whose cellular basis is not fully understood. Among these, the AEC syndrome is characterized by severe skin defects at birth with inflammation, epidermal fragility and decreased skin barrier function [36]. We have found that ΔNp63 mutants carrying natural mutations found in AEC syndrome patients cannot activate *Cldn-1* expression anymore and act as dominant negative molecules towards the wild type ΔNp63α isoform. This latter observation is in agreement with the heterozygous nature of AEC syndrome [37]–[39]. On the contrary, ΔNp63 mutants derived from SHFM-IV syndrome, which is not associated to skin fragility, do not display any defect in the activation of *Cldn1* reporter gene, indicating *Cldn-1* as an AEC specific p63 target gene. To verify this hypothesis we have analyzed Cldn-1 expression by immunohistochemistry in the lesional skin of a patient affected by AEC syndrome, who was genotyped as having the I537T mutation in the SAM domain of p63. We have observed a strong downregulation of Cldn-1 levels in basal and suprabasal layers of AEC epidermis as compared to normal human skin. p63 expression was not affected, further reinforcing the concept of dominant negative nature of AEC mutations.

Taken together our data indicate *Cldn-1* as transcriptional target of the disease gene *p63*, potentially involved in the pathogenesis of the skin dysplasia observed in AEC patients.
